# From diagnostic errors to diagnostic excellence in emergency care: Time to flip the script

**DOI:** 10.1111/acem.15033

**Published:** 2024-10-20

**Authors:** Prashant Mahajan

**Affiliations:** ^1^ Section of Pediatric Emergency Medicine, Department of Emergency Medicine University of Michigan Ann Arbor Michigan USA

The National Academies of Sciences, Engineering, and Medicine (NASEM) highlighted the substantial patient safety and economic impact of diagnostic errors in their report, “Improving Diagnosis in Health Care.” Diagnostic error is defined by the committee as “a failure to establish an accurate and timely explanation of the patient's health problem(s) or communicate that explanation to the patient.”^1^ If we accept this definition, then emergency departments (EDs) are arguably the only settings where the NASEM framework applies to nearly every patient–provider encounter. With ~140 million annual ED visits,^2^ ED providers make critical gatekeeping decisions regarding the safest and most appropriate ways to care for each patient with far‐reaching implications for both individuals and the entire health care system. EDs also serve as the sole access point to health care acting as a safety net for the most vulnerable among us, namely the indigent, uninsured, elderly, children, and minority communities. However, EDs face challenges such as overutilization, decreased functional capacity from admitted patients awaiting an inpatient bed, and financial pressures leading to closures. The NASEM concluded that the nation's emergency medical system is overburdened, underfunded, and highly fragmented.^3^ Unfortunately, emergency care delivery is subject to contradictory perceptions: being praised as heroic, especially during the COVID‐19 pandemic, and criticized as a site of inappropriate, incomplete, unnecessary, unsafe, and costly care. A recent report suggests that approximately one in 18 ED visits (~7.5 million) result in diagnostic error, and nearly one in 50 ED visits (~2.6 million) experience an adverse event, making the ED a prime laboratory for studying diagnostic safety.^4^ However, it should be noted that the veracity of these numbers is questionable as they are based on suboptimal and incomplete evidence. Regrettably, incomplete scientific findings are highlighted by the media, eroding patients’ trust in ED care. Additionally, long ED wait times contribute to avoidable undesirable outcomes, negatively impact the patient experience, and further reinforce the perception of subpar care. The aim of this paper is to describe the unique challenges to accurate and timely diagnostic decision making in the ED and identify opportunities to mitigate them.

## WHY IS DIAGNOSTIC DECISION MAKING CHALLENGING IN THE ED?

The ED is a challenging environment because decisions are made on patients without preexisting relationships to the providers under substantial time and information constraints. Illnesses of varying severity are often evolving in real time or in response to lifesaving interventions. Studies reveal that in ~80% of encounters, ED providers generated the first diagnostic hypothesis prior to patient evaluation and made most of their differential diagnoses within seconds of entering the patient's room.^5^ Although this fast‐thinking approach explained under the dual process theory is needed and largely successful, it is prone to unconscious and unrecognized bias including but not limited to anchoring on a diagnosis despite evidence to the contrary. Decision fatigue, frequent interruptions, and multiple handoffs that lead to communication gaps between providers of varying skillset and subspecialities contribute to diagnostic errors. Provider‐level factors such as lack of sleep and system‐level factors such as overcrowding and deteriorating work environment are directly responsible for ED providers feeling a high level of burnout, estimated at 60% and the highest among all specialties, which further contributes to suboptimal diagnostic outcomes.^6^

In addition to this challenging context, research on diagnostic decision making itself remains incomplete. For instance, we do not fully understand the process of clinical reasoning. As a result, interventions that require clinicians to use analytical and deductive reasoning, along with metacognitive (thinking about thinking) and debiasing strategies, have proven ineffective.^7^ Studies have shown that clinicians are unreliable in identifying their own biases and often exhibit overconfidence, making it difficult for them to recognize when their diagnosis was incorrect.^8^, ^9^ Additionally, progress in diagnostic safety research is impeded by the absence of a unified taxonomy for defining errors, contributory factors, and the severity of harm. Studies on the epidemiology of diagnostic events are inherently retrospective, subject to hindsight bias and thus unreliable, as even experts may disagree on whether an error has occurred.^1^ The current approaches to improving diagnostic quality do not align well with the unique health care delivery in the ED. Despite increasing interest in research on diagnostic errors, only a few interventions have been successfully developed and tested in real‐world settings. Therefore, it is crucial for the ED community to change the narrative on diagnostic errors.

## HOW CAN WE “FLIP THE SCRIPT” AND MOVE AWAY FROM LABELING EVENTS AS ERRORS AND FOSTER A CONSTRUCTIVE CULTURE OF DIAGNOSTIC EXCELLENCE IN THE ED?

To improve diagnostic safety and change the narrative, several steps must be taken. The first step involves acknowledging that the diagnostic process consists of three main groups: the patient and their caregivers, health care providers (including physicians, nurses, and other team members), and the health care system. Diagnostic mishaps can occur because of a complex interaction of various factors. These factors include patient‐related issues, such as altered mental state or language barriers, as well as provider‐related factors like cognitive biases and gaps in education. Additionally, system‐related factors, such as overcrowding, the practice of boarding inpatients in the ED, and a poor safety culture, can also contribute to diagnostic errors. It is important to note that these mishaps are rarely attributed to a single individual or factor.^1^

**FIGURE 1:**
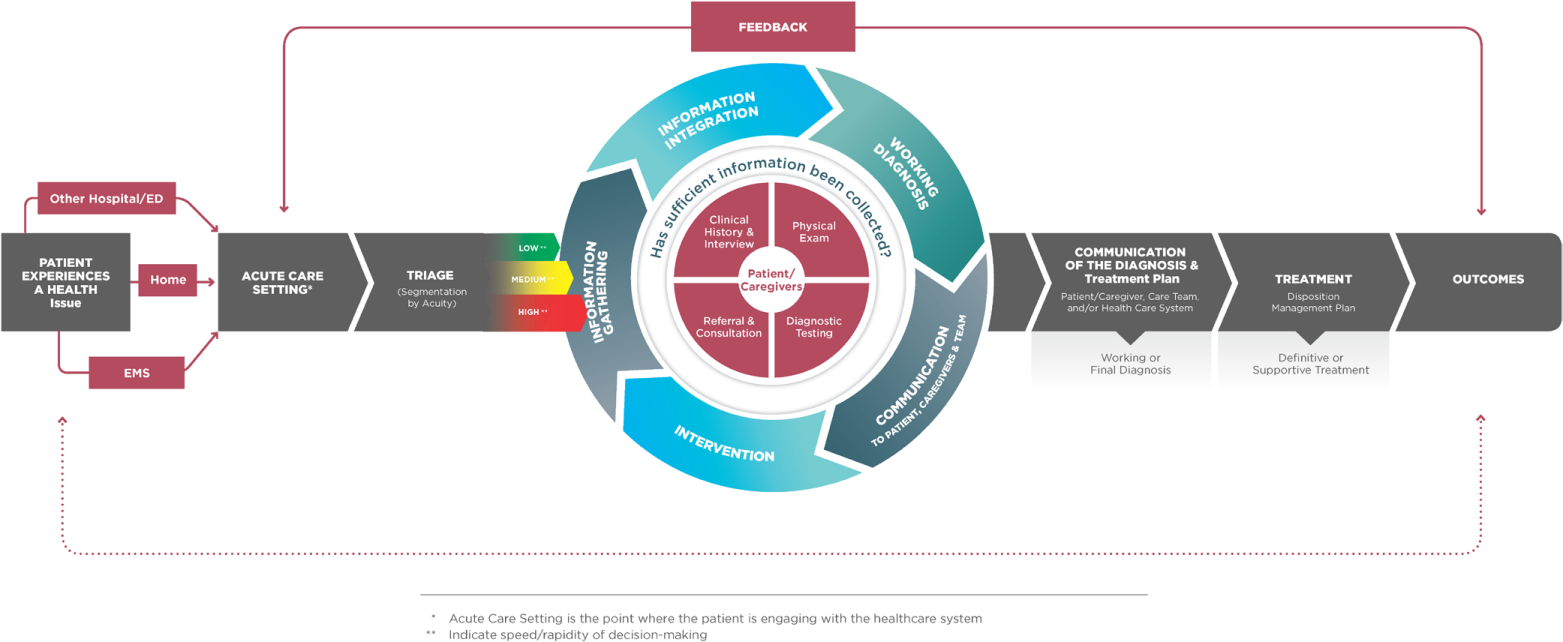
Diagnostic Process in the Emergency Department: An Adaptation of NASEM Framework.

The second step involves a recognition that the ED is just one part of the entire diagnostic journey, which can extend from the prehospital setting to inpatient care and ultimately back to the community. Thus, the NASEM diagnostic process framework needs to be adjusted to better align with how health care is delivered in the ED.^10^ One crucial aspect to consider is the prehospital phase, which can be seen as the starting point or “time zero” of the diagnostic process and can have a significant impact. Factors such as the mode of arrival and the source of referral can introduce bias by influencing the perceived severity of the disease or altering the clinical presentation due to interventions that occurred prior to the ED encounter. An important improvement could consider the implementation of a consistent feedback process for ED providers, informing them about the ultimate outcomes of their patients and the accuracy of their diagnoses (Figure 1).

The third step involves learning from the more common successful diagnostic outcomes in addition to investigating diagnostic failures. This approach, known as Safety‐II, recognizes the dynamic and unpredictable nature of health care.^11^ Patients, providers, and systems continuously adapt to deliver high‐quality care. Adaptation is the key factor that results in a gap between “work as imagined” (i.e., written policy) and “work as done” (i.e., actual practice). It also allows patients to contribute and clinicians to handle unexpected situations (adaptive safety). Research in this area should focus on understanding how adaptations occur across a range of ED presentations, settings (academic ED or a community ED), and demographic factors of both patients and providers (age, race, ethnicity, sex, language, and appearance). These factors can shed light on biases and the impact of social determinants of health.

The fourth step involves advocating for diagnostic safety research with ongoing and increasing support from federal agencies, specifically AHRQ and PCORI. It is imperative that research incorporates the simultaneous and deliberate perspectives of patients, providers, and systems in all aspects of diagnostic safety research, ranging from proof‐of‐concept studies to translation to patients, practice, and populations. Collaboration from all stakeholders is needed to co‐design research questions, identify relevant study designs, co‐create interventions, and assess their impact. Furthermore, a multidisciplinary approach that includes, but is not limited to, experts in cognitive science, health care education, clinical informatics, and human factors engineering will be essential to decipher the adaptive aspects of diagnostic decision making in the real world.

Finally, advances in data science—especially the recent explosion of artificial intelligence algorithms—hold substantial potential for performing tasks such as learning, aiding decision making, and possibly reasoning in the future. A pragmatic approach to implementing such powerful technologies must be balanced with a clear understanding of the limitations of current models, which include worsened bias, patient privacy concerns, and the exacerbation of the digital divide across economically and socially disadvantaged populations.^12^

In summary, reframing diagnostic errors as diagnostic excellence aligns with NASEM's guidance to promote a nonaccusatory, nonpunitive culture that encourages open discussion of safety events without fear of legal or disciplinary measures. Emergency providers need to advocate for their specialty and collaborate with patients and health system representatives for EDs to serve as a beacon for research and implement safe, effective, patient‐, provider‐, and system‐centered interventions to mitigate harm and promote diagnostic excellence.

## CONFLICT OF INTEREST STATEMENT

The author declares no conflicts of interest.
